# Apraxia and Motor Dysfunction in Corticobasal Syndrome

**DOI:** 10.1371/journal.pone.0092944

**Published:** 2014-03-24

**Authors:** James R. Burrell, Michael Hornberger, Steve Vucic, Matthew C. Kiernan, John R. Hodges

**Affiliations:** 1 Neuroscience Research Australia, Sydney, New South Wales, Australia; 2 Prince of Wales Clinical School Hospital, Sydney, New South Wales, Australia; 3 University of New South Wales, Sydney, New South Wales, Australia; 4 Sydney Medical School, University of Sydney, Sydney, New South Wales, Australia; University of Bologna, Italy

## Abstract

**Background:**

Corticobasal syndrome (CBS) is characterized by multifaceted motor system dysfunction and cognitive disturbance; distinctive clinical features include limb apraxia and visuospatial dysfunction. Transcranial magnetic stimulation (TMS) has been used to study motor system dysfunction in CBS, but the relationship of TMS parameters to clinical features has not been studied. The present study explored several hypotheses; firstly, that limb apraxia may be partly due to visuospatial impairment in CBS. Secondly, that motor system dysfunction can be demonstrated in CBS, using threshold-tracking TMS, and is linked to limb apraxia. Finally, that atrophy of the primary motor cortex, studied using voxel-based morphometry analysis (VBM), is associated with motor system dysfunction and limb apraxia in CBS.

**Methods:**

Imitation of meaningful and meaningless hand gestures was graded to assess limb apraxia, while cognitive performance was assessed using the Addenbrooke's Cognitive Examination – Revised (ACE-R), with particular emphasis placed on the visuospatial subtask. Patients underwent TMS, to assess cortical function, and VBM.

**Results:**

In total, 17 patients with CBS (7 male, 10 female; mean age 64.4+/− 6.6 years) were studied and compared to 17 matched control subjects. Of the CBS patients, 23.5% had a relatively inexcitable motor cortex, with evidence of cortical dysfunction in the remaining 76.5% patients. Reduced resting motor threshold, and visuospatial performance, correlated with limb apraxia. Patients with a resting motor threshold <50% performed significantly worse on the visuospatial sub-task of the ACE-R than other CBS patients. Cortical function correlated with atrophy of the primary and pre-motor cortices, and the thalamus, while apraxia correlated with atrophy of the pre-motor and parietal cortices.

**Conclusions:**

Cortical dysfunction appears to underlie the core clinical features of CBS, and is associated with atrophy of the primary motor and pre-motor cortices, as well as the thalamus, while apraxia correlates with pre-motor and parietal atrophy.

## Introduction

Corticobasal syndrome (CBS) is a neurodegenerative disorder characterized by a combination of cognitive deficits and multi-faceted motor system dysfunction,[1]–[4] with asymmetric rigidity, bradykinesia, and prominent asymmetric limb apraxia.[5] In addition, cognitive dysfunction is now recognized as a core clinical feature of CBS. Patients typically develop progressive disturbances of language or behavior, which overlap with those seen in frontotemporal dementia (FTD). Unlike other patients within the FTD spectrum, visuospatial dysfunction is characteristic,[4], [6]–[8] and has been included as a component of most clinical diagnostic criteria for CBS.[9]–[11]

Transcranial magnetic stimulation of the motor cortex has been used to explore motor system dysfunction in CBS. Previous studies have demonstrated altered resting motor threshold (RMT) [12], [13] and reduced short-interval intracortical inhibition (SICI).[12]–[15] These abnormalities have been attributed to motor cortex dysfunction, although the role of concomitant basal ganglia dysfunction remains unclear. The relationship of cortical dysfunction to clinical symptoms and pathology has not been studied.

Although apraxia may be seen in other neurodegenerative diseases,[16], [17] the severity of asymmetric limb apraxia appears to be distinctive in CBS [2], [3] and constitutes an important diagnostic feature.[9]–[11] Apraxia may be defined as the inability to perform a motor task, despite intact power, sensation, coordination, comprehension and cooperation [18] and is typically the earliest symptom in CBS.[3] The classification of limb apraxia is complex, with distinctions made between transitive (involving tool use) and intransitive (actions not requiring tools, such as “waving”) actions, or subdivision of apraxia into ideomotor, ideational, and limb-kinetic types.[18] Some forms of apraxia are associated with left hemisphere pathology, whereas others may be associated with right hemisphere damage.[18]–[20] Furthermore, some apraxia subtypes have been attributed to parietal pathology,[21], [22] but other structures including the motor cortex (primary and supplementary) and the basal ganglia, have also been implicated.[17], [19], [21] Atrophy of the primary motor cortex, basal ganglia, or parietal lobe is common in CBS,[23], [24] suggesting that dysfunction of these regions may contribute to the development of apraxia in the syndrome.

A range of pathologies may present as CBS. Initial reports emphasized an underlying tauopathy (referred to pathologically as corticobasal degeneration), with similar features to those seen in many cases of frontotemporal lobar degeneration. More recently Alzheimer's disease, TAR DNA-binding protein 43 intraneuronal inclusions, and progressive supranuclear palsy have been reported in cases of CBS.[4], [23]–[26] The pattern of cortical atrophy varies markedly in CBS, depending on the underlying pathology,[24], [27] but whether the pattern of atrophy explains the frequency and severity of apraxia or motor system dysfunction in CBS is unknown.

Although the pattern of cerebral atrophy in CBS varies significantly, previous studies have consistently demonstrated atrophy of the primary motor cortex.[24], [27] This finding is consistent with reports of motor cortex dysfunction studied using transcranial magnetic stimulation,[12]–[15] although the relationship between physiological changes and cerebral atrophy has not been investigated. Whether primary motor cortex involvement contributes to apraxia is not known.

The present study explored several hypotheses; firstly, that limb apraxia in CBS – in part – reflects impaired visuospatial processing. Secondly, that motor system dysfunction can be demonstrated in CBS, using paired-pulse threshold tracking transcranial magnetic stimulation, and that motor system dysfunction and limb apraxia both reflect pathological involvement of cortical and subcortical motor structures. Finally, that atrophy of the primary motor cortex, studied using voxel-based morphometry analysis (VBM), is associated with motor system dysfunction and limb apraxia in CBS.

## Materials and Methods

### Study Participants

Patients with a clinical diagnosis of CBS were recruited consecutively from a specialist cognitive disorders clinic. The study was approved by the South Eastern Sydney Local Health District Human Research Ethics Committee and performed after written informed consent was obtained from all participants. In accordance with PLoS One policies, the data from the present study may be made available on request. The diagnosis of CBS was established through a detailed clinical assessment and neuropsychological evaluation, and all patients met recent diagnostic criteria.[11] Structural imaging with magnetic resonance imaging was also performed, but the results were not used to select or exclude patients for the present study. Patients with other alternative diagnoses notably idiopathic Parkinson's disease, progressive supranuclear palsy, vascular dementia, or psychiatric disease were also excluded.

In total, 34 participants were included in the study; 17 with CBS and 17 control subjects. Of the 17 patients with CBS, 41.2% were male and the mean age at assessment was 64.4+/−6.6 years. The mean symptom duration was 54.6+/−18.0 months. A database of volunteers was used to recruit control subjects. Each individual patient was matched to a control subject of the same gender (male gender in 41.2%). Where possible CBS patients were matched to a control subject of the same age in years. In practice, control subject age was matched to within 2 years of CBS patient age in all but two cases, in which the age difference was 3 and 4 years respectively. The mean age of control subjects was 64.4+/−7.3 years and this was not significantly different from that of CBS patients (P = 1.0). Controls and CBS patients were not specifically matched for handedness. Control subjects were included if they had no history of neurological diseases such as dementia, stroke, multiple sclerosis, Parkinson's disease or other movement disorders. Controls who demonstrated cognitive impairment on neuropsychological evaluation or incidental abnormalities on MRI scanning were excluded from the study.

### Clinical Assessment

Apraxia was systematically assessed using a semi-structured approach. Specifically, patients were asked to imitate 4–5 meaningful (for example; the “Thumb's up” or “A-Okay” gestures) and 4–5 meaningless hand gestures, using both the right and left hands,[28] to allow a judgment of the severity of limb apraxia. The assessments were videotaped and the overall level of apraxia in the right and left upper limbs was graded on a 4 point scale (no apraxia  = 0, mild  = 1, moderate  = 2, severe  = 3) independently by two examiners (JRH and JRB). Mild (score  = 1) apraxia was defined as occasional/minor errors or hesitancy with the correct target gesture achieved. Moderate (score  = 2) apraxia was defined as consistent errors of hand position, but with some target gestures achieved. Severe (score  = 3) apraxia was defined as the inability to achieve any target accurately. Using this grading system, the overall rater scores did not differ significantly between individual raters (P = 0.154, see Table S1). Furthermore, there was a strong and highly significant correlation between scores from both raters (P = 0.005, Spearman's coefficient 0.75). Finally, inter-rater reliability analyses, performed using the intraclass correlation coefficient, revealed excellent convergence between the two raters; overall score (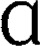 = 0.848). Similarly, patients were asked to imitate 2–3 oro-buccal gestures (for example; “lick your lips” or “cough”) to judge the severity of oro-buccal apraxia. Individual examiner ratings from the right (0–3) and left (0–3) upper limbs were averaged to produce scores for limb-meaningful (0–6 points), limb-meaningless (0–6 points), and the oro-buccal score was added to produce an overall apraxia score (0–15 points), with higher scores indicating a greater degree of apraxia. Other components of apraxia – such as imaginary tool usage (for example; “brush your teeth” or “comb your hair”) were also included in the assessment, but were not graded consistently (i.e. formal grading was not completed on all patients). These components of the assessment were therefore excluded from the analysis and were not used to calculate the apraxia scores. Imitation, rather than pantomime (i.e. production of meaningful gestures from memory) was chosen, as imitation may be more sensitive to disturbances of praxis in CBS.[29], [30]

All patients underwent a standardized clinical assessment by a single examiner (JRB) to detect clinical evidence of motor system dysfunction such as weakness and hyperreflexia.[31] Muscle power was graded according to the Medical Research Council grades, after taking into account the degree of limb apraxia, and individual muscle scores were added to calculate the Medical Research Council sum-score (MRCSS) for each patient. Hyperreflexia, defined as exaggerated deep tendon reflexes elicited with minimal stimulus, or pathological spread of reflexes, was recorded in each patient.

Since a disease specific functional rating scale has not been developed for CBS, the Amyotrophic Lateral Sclerosis Functional Rating Scale – Revised (ALSFRS-R),[32] was chosen as a validated measure of motor functional capacity.[33] The ALSFRS-R comprises 12 questions regarding ability to perform everyday tasks; each response is graded from 0–4 with functional impairment reflected by reduced scores. In addition to ALSFRS-R totals, bulbar, fine motor, gross motor and respiratory ALSFRS-R sub-scores were also calculated for each patient.

### Cognitive testing

Cognitive screening was performed using the Addenbrooke's Cognitive Examination – Revised (ACE-R).[34], [35] The ACE-R is a brief cognitive screening tool that includes an assessment of five cognitive domains, and has been used to evaluate cognitive dysfunction in CBS previously.[8] The domains examined by the ACE-R include: attention (18 points), memory (26 points), verbal fluency (14 points), language (26 points) and visuospatial ability (16 points). The ACE-R is scored out of 100 points and a score at or below 88/100 detects dementia with a sensitivity of 94% and specificity of 89%.[35] The visuospatial component of the ACE-R consists of tasks that require the use of a pencil (e.g. copy of interlocking pentagons, drawing of a clock-face), as well as tasks that simply require interpretation of visual information (e.g. dot counting, partial letter recognition). Marked limb apraxia might be expected to impair performance on tasks requiring manipulation of a pencil, particularly if the dominant hand is maximally impaired. With this in mind, performance on both subcomponents of the ACE-R visuospatial task, designated as “written” and “visual” visuospatial subcomponents (each scored out of 8 points), were analyzed independently.

### Neurophysiological Assessments

The paired pulse, threshold-tracking transcranial magnetic stimulation protocol was used to assess cortical function in CBS patients and control subjects.[36]–[38] This testing protocol was preferred over the constant stimulus paired pulse technique as the motor evoked potential amplitude often varies significantly from stimulus to stimulus thereby necessitating multiple stimuli, with subsequent averaging, at each level of stimulus intensity.[39] The threshold-tracking paradigm overcomes this potential limitation by tracking a target response of 0.2 mV, which lies in the middle of the linear logarithmic stimulus-response relationship over a hundred-fold range of responses from about 0.02 to 2 mV.[40] As such, larger variations in the MEP amplitude would translate to smaller variations in the stimulus intensity (the outcome variable), potentially enabling more accurate recordings of TMS parameters. Specifically, using the paired-pulse threshold tracking protocol stimuli are repeated until the target response has been achieved and averaging of multiple responses is not required. This technique has been successfully used in frontotemporal dementia,[31] as well as motor neuron disease and related disorders.[33], [36]–[38], [41]

**Figure 1:**
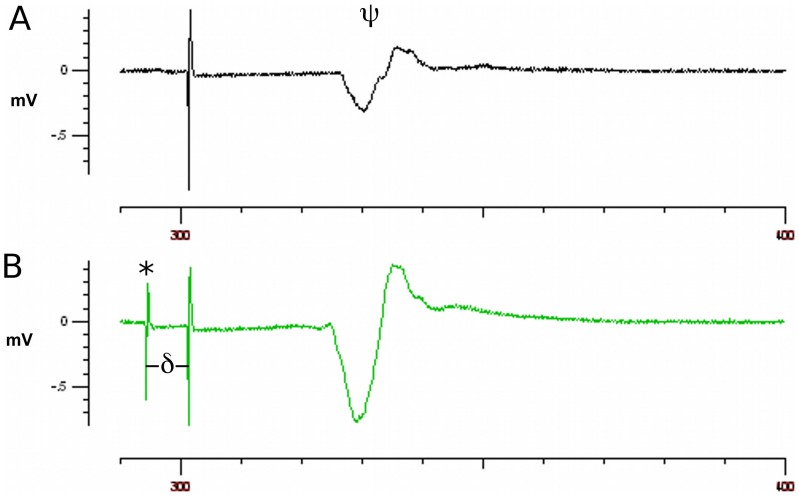
The paired-pulse transcranial magnetic stimulation protocol. (**A**) Motor evoked potentials (¥) were recorded from the abductor pollicis brevis muscle following magnetic stimulation of the motor cortex. The stimulus intensity required to achieve a target response of 0.2 mV, defined as the resting motor threshold (RMT), was determined following a single magnetic stimulus. (B) Pairs of pulses were then delivered; a conditioning impulse with an intensity set at 70% of RMT (*) followed by a test impulse which varied in intensity in order to maintain the target response of 0.2 mV. As the protocol proceeded, the interval between the two stimuli – defined as the interstimulus interval (∂) – was varied from 1–20 ms. SICI was defined as the increase in test impulse intensity (i.e. test - RMT) required to achieve the target response at interstimulus intervals of 1–7 ms, represented as a percentage of RMT. *Please note, this figure (A) is simply intended to illustrate how the RMT is determined and the response reproduced here does not necessarily represent an accurate measurement of RMT. Furthermore, the intensities of the stimuli used in A and B differ; therefore the larger amplitude motor response in B does not necessarily reflect intracortical facilitation following the conditioning impulse.*

According to the threshold tracking protocol, the motor cortex was stimulated using magnetic pulses delivered via a 90 mm magnetic circular coil placed over the subject's scalp, and the resultant motor evoked potentials were recorded from the abductor pollicis brevis muscle in the hand at rest. Patients were repeated asked to relax the hand being tested and the protocol was recommenced if voluntary or involuntary (e.g. mild dystonia) motor activity interfered with electromyographic silence. By default, the right hand was used for transcranial magnetic stimulation studies; if a stable response could not be obtained on the right, the left hand was studied. If a stable motor response could not be obtained on either side, despite maximal stimulus intensity, the motor cortex was classified as relatively inexcitable and the protocol was ceased. In such cases, no subsequent measures of cortical excitability could be determined. The optimal coil position, defined, as the position that elicited the most stable motor response, was determined first (See Figure 1A), followed by the resting motor threshold (RMT). The RMT was defined as the amount of stimulus required to evoke the target motor evoked potential of 0.2 millivolts (mV). Provided a stable motor response was obtained with single magnetic stimuli, the paired pulse component of the protocol was initiated. In order to deliver pairs of pulses, two high-power magnetic stimulators were connected via a BiStim device (Magstim Co., Whitland, South West Wales, UK) and used to deliver paired stimuli that could be set independently and delivered through one coil. The first stimulus – the conditioning impulse – was delivered at an fixed intensity of 70% RMT, and the second stimulus – the test impulse – was varied in intensity to achieve the target motor evoked potential of 0.2 mV (See Figure 1B).

As the protocol proceeded, the time between conditioning and test impulses – referred to as the interstimulus interval – was varied from 1 to 20 milliseconds (ms). The motor responses were amplified and filtered (3 Hz–3 kHz) using a GRASS ICP511 AC amplifier (Grass-Telefactor, Astro-Med Inc., West Warwick, RI, USA) and sampled at 10 kHz using a 12-bit data acquisition card (National Instruments PCI-MIO-16E-4). The protocol was driven by QTRACS software (Institute of Neurology, Queen Square, London, UK).

In normal individuals, increased test impulse intensity is required to produce the target motor response following a conditioning impulse when the interstimulus interval is between 1–7 ms. This phenomenon, referred to as short interval intracortical inhibition (SICI), reflects relative cortical inhibition induced by the conditioning impulse at short interstimulus intervals. In the present study SICI was defined as the increase in test impulse intensity (i.e. test - RMT) required to achieve the target response at interstimulus intervals of 1–7 ms, represented as a percentage of RMT.[36], [40] Peak SICI was recorded at an interstimulus interval of 3.5 ms and average SICI was calculated as the mean of SICI values recorded at each interstimulus interval from 1–7 ms. In addition, the maximal motor evoked potential amplitude and minimum motor evoked potential latency were recorded, and the maximal cortical silent period was calculated. The maximal cortical silent period was defined as the duration of electrical silence recorded from the abductor pollicis brevis induced by sustained, sub-maximal muscle contraction, following single pulse magnetic stimulation.[42] The central motor conduction time was calculated using the F-wave method.[43], [44] The compound motor action potential amplitude following electrical stimulation of the median nerve at the wrist was recorded in millivolts (mV) from the abductor pollicis brevis muscle in the hand using 5-mm Ag-AgCl surface electrodes (ConMed, Utica, USA). The motor evoked potential amplitude was also expressed as a percentage of compound motor action potential amplitude.

### Voxel-Based Morphometry

All 17 CBS patients and 17 age-matched healthy controls underwent magnetic resonance imaging (MRI) according to a standardized protocol using a 3-Tesla Phillips MRI scanner with standard quadrature head coil (8 channels). The 3D T1-weighted images were acquired with the following parameters: coronal orientation, matrix 256×256, 200 slices, 1×1 mm2 in-plane resolution, slice thickness 1 mm, TE/TR  = 2.6/5.8 ms, and TFE/FFE Pulse sequence.

3D T1-weighted sequences were used to perform a VBM analysis [45], [46] using the Functional Magnetic Resonance Imaging of the Brain (FMRIB) Software Library (FSL) software package [47] (see http://www.fmrib.ox.ac.uk/fsl/fslvbm/index.html). Tissue segmentation was carried out using FMRIB's Automatic Segmentation Tool (FAST) [48] from brain-extracted images. The resulting grey matter partial volume maps were aligned to the Montreal Neurological Institute standard space (MNI152) using the nonlinear registration approach via FMRIB's Nonlinear Image Registration Tool (FNIRT),[49], [50] which uses a b-spline representation of the registration warp field.[51] The registered partial volume maps were then modulated (to correct for local expansion or contraction) by dividing them by the Jacobian of the warp field. The modulated images were smoothed with an isotropic Gaussian kernel with a standard deviation of 3 mm (Full Width at Half Maximum: 8 mm). Finally, a voxel-wise general linear model (GLM) was applied and permutation-based non-parametric testing was used to form clusters with the Threshold-Free Cluster Enhancement (TFCE) method.[52] Multiple comparison corrections were not performed as these often exclude meaningful results in covariance analyses, which correlate variables (i.e. RMT, SICI) with atrophy rather than compare groups. Instead, uncorrected P values were used. To reduce the likelihood of false positive correlations a more a stringent significance level of P<0.001 and a contiguous cluster threshold of 20 voxels were applied.[53]–[55]

### Statistical Analysis

Statistical analysis was performed by a single author (JRB) and carried out using the Statistical Package for Social Sciences (version 19.0, SPSS Inc.; Chicago, IL, USA). The Wilcoxon signed rank test was used to compare related measures (e.g. limb-meaningful and limb-meaningless apraxia scores) in individual patients, as well as compare apraxia scores between different independent raters. Comparisons of neurophysiological data were first made between patients with CBS and control subjects. Subjects were later grouped according to their RMT; patients with an RMT <50% (RMT <50%), patients with an RMT >50% (RMT >50%), and patients with an inexcitable motor cortex (inexcitable), and the three groups were compared. Neurophysiological parameters could not be determined in patients with an inexcitable motor cortex, so these patients were excluded from several subsequent analyses. In excitable patients, an RMT of 50% was used to define the groups as this approximated the median recorded RMT for the patients in the study. Continuous variables were analyzed using analysis of variance (ANOVA) when normally distributed or the Kruskal–Wallis test when non-normally distributed. Pair-wise comparisons were performed using the student's t test when the data was normally distributed and the Mann-Whitney test when non-normally distributed. Categorical data were analyzed using the Chi-Square test. Correlations between continuous variables were performed using Spearman correlation for non-normally distributed samples, after application of the Bonferroni correction,[56] with a P-value of <0.05 regarded as significant. Partial correlation was used to control for potential confounding variables such as age and symptom duration.

## Results

As mentioned, 34 participants were included in the study; 17 with CBS and 17 age and gender matched controls. Of patients with CBS, 7 (41.2%) were male. The mean age at assessment was 64.4+/−6.6 years and the mean symptom duration was 54.6+/−18.0 months. All but one CBS patient was right-hand dominant. The maximally apraxic hand was the right hand in 10 CBS patients, the left hand in 4 patients, and both hands were equally affected in 3 patients. The single left-hand dominant patient had bilateral limb apraxia. The right hand was studied in 11 (84.6%) CBS patients with an excitable motor cortex, whereas the left hand was used in 2 (15.4%) other patients. Motor dysfunction in CBS patients was characterized by rigidity and bradykinesia, rather than tremor (see Table S2). Postural instability was present in 9 (52.9%) CBS patients, but myoclonus and alien limb phenomenon were uncommon, each present in only 3 (17.6%) patients. Further details regarding the clinical presentation of CBS patients are presented in Table 1.

**Table 1:** Characteristics of Individual CBS patients.

Patient	Age at assessment (years)	Gender	Symptom duration (months)	ACE Total (100 points)	ACE Visuospatial (16 points)	Overall apraxia (0–15)	Clinical Presentation	MRI findings
**1**	66	Male	36	62	12.0	11.0	Right sided limb apraxia initially, later developed language disturbance	Generalised atrophy, with symmetrical frontal, temporal, parietal and occipital lobe involvement.
**2**	57	Male	60	53	5.0	7.0	Cognitive dysfunction with executive impairment, visuospatial dysfunction and later developed marked left sided limb apraxia	Generalised atrophy with particular involvement of frontal, temporal (left > right), parietal and occipital lobes bilaterally.
**3**	62	Female	48	84	14.0	3.0	Speech hesitancy and deterioration in hand writing, later developed right sided limb apraxia	Frontal, temporal (left > right) and parietal atrophy.
**4**	56	Female	70	25	2.0	11.5	Cognitive dysfunction with executive impairment and visuospatial deficits initially, followed by bilateral limb apraxia and language disturbance	Frontal atrophy bilaterally, particularly involving Broca's area, and a degree of parietal atrophy.
**5**	68	Female	36	91	16.0	6.5	Marked left sided limb apraxia and rigidity, with subtle language dysfunction	Frontal and parietal atrophy bilaterally.
**6**	68	Female	36	84	10.0	6.5	Executive dysfunction with some disorientation and difficulty writing, followed by left sided limb apraxia	Frontal atrophy, particularly involving the medial frontal lobes.
**7**	59	Female	72	86	12.0	6.0	Right sided limb apraxia, with spelling errors on hand writing, executive dysfunction, and visuospatial deficits	Inferior frontal and parietal atrophy (left >right).
**8**	79	Male	36	78	15.0	2.5	Progressive language disturbance with subsequent development of right upper limb apraxia	Bilateral frontal atrophy, with particular involvement of the left peri-insular region.
**9**	72	Female	83	86	15.0	3.5	Difficulty using the right leg and hand, with mild visuospatial deficits	Bilateral frontal, temporal, and parietal atrophy.
**10**	61	Female	60	*	*	14.0	Executive dysfunction and deterioration in handwriting, with right sided limb apraxia	Generalised atrophy (left > right).
**11**	70	Female	48	30	4.0	12.5	Progressive right sided limb apraxia, with subsequent executive, visuospatial and language disturbance	Frontal, temporal, and parietal atrophy (left > right).
**12**	57	Male	21	77	14.0	6.0	Problems with calculation and hand-writing, followed by language disturbance and bilateral limb apraxia	Left parietal atrophy.
**13**	59	Male	84	86	13.0	3.0	Disturbance of handwriting, followed by right sided limb apraxia and language disturbance	Left inferior frontal and peri-insular atrophy.
**14**	65	Male	60	38	6.0	5.0	Progressive language disturbance and difficulty writing, followed by right sided apraxia	Frontal and parietal atrophy.
**15**	66	Male	48	38	5.0	11.5	Language disturbance and difficulty with hand-writing, followed by right sided rigidity and apraxia	Bilateral frontal and parietal atrophy.
**16**	72	Female	70	24	6.0	11.0	Difficulty with writing and language disturbance, followed by right sided limb apraxia	Parietal and temporal atrophy (left > right), with particular involvement of Broca's area and the left mesial temporal lobe.
**17**	58	Female	60	79	8.0	12.5	Difficulty writing and right sided limb apraxia, with subsequent language impairment	Bilateral frontal and parietal atrophy (right > left).

Patients commonly presented with a mixture of limb symptoms and cognitive dysfunction, with limb apraxia and visuospatial dysfunction featuring prominently. Other cognitive deficits included language disturbance (often with early problems in hand-writing) and executive dysfunction. * ACE-R abandoned due to severity of cognitive deficit.

Patients with CBS had moderate motor functional disability, due predominantly to limb apraxia, parkinsonism, and rigidity, rather than weakness (see Table 2). All patients had difficulty producing meaningful and meaningless hand gestures, reflected by increased limb-meaningful and limb-meaningless apraxia scores, while 6 of 16 (37.5%) of CBS patients had evidence of oro-buccal apraxia, albeit relatively mild as reflected in the oro-buccal apraxia subscore (0.7+/- 0.9). There was no significant difference between the mean limb-meaningful (3.6+/−1.8) and limb-meaningless (3.9+/−1.9, P = 0.203) scores in CBS patients, while dystonia was identified in 5 (29.4%) patients. CBS patients were functionally impaired, reflected in markedly reduced fine and gross motor ALSFRS-R sub-scores, despite normal limb power in all but one patient.

**Table 2:** Clinical Characteristics of CBS patients.

	CBS
Number of Patients	17
Male	7 (41.2%)
Age	64.4+/−6.6
Symptom Duration (months)	54.6+/−18.0
Hyperreflexia (% patients)	12 (70.6%)
Weakness (% patients)	1 (6.3%)
MRCSS Total (0–60)	59.8+/−1.0
**Apraxia**	
- Oro-buccal Apraxia (0–3)	0.7+/−0.9
- Limb-Meaningful (0–6)	3.6+/−1.8
- Limb-Meaningless (0–6)	3.9+/−1.9
- Overall Apraxia Score (0–15)	7.8+/−3.9
**ALSFRS-R**	
- Bulbar	10.7+/−1.4
- Fine Motor	5.7+/−3.4
- Gross Motor	8.5+/−2.9
- Respiratory	11.8+/−0.5
- Total	36.7+/−6.2
**ACE-R**	
- Attention (18 points)	13.1+/−5.7
- Memory (26 points)	15.5+/−8.7
- Fluency (14 points)	5.4+/−4.3
- Language (26 points)	17.2+/−7.7
- Visuospatial (16 points)	9.2+/−5.1
- Total (100 points)	60.1+/−28.7
**MMSE**	19.7+/−9.3

CBS patients had marked functional impairment with reduced ALSFRS-R fine motor and gross motor sub-scores, despite normal limb power. CBS patients were at least moderately cognitively impaired, with deficits in multiple cognitive domains including visuospatial function. MRCSS  =  medical research council sum score, ALSFRS-R  =  amyotrophic lateral sclerosis functional rating score – revised, ACE-R  =  Addenbrooke's cognitive examination – Revised, MMSE  =  mini-mental status examination.

Patients with CBS performed poorly on the visuospatial component of the ACE-R (Table 2 and Table S3). As might have been expected, CBS patients performed better on the “visual” subcomponent (6.2+/−2.0) than on the “written” subcomponent of the ACE-R (3.2+/−3.0, P = 0.001, see *Methods*). However, regardless of whether the right limb (dominant in all but 1 patient) or left limb was maximally apraxic, there was no significant difference in performance on the “visual” or “written” subcomponents of the ACE-R, suggesting that poor performance on visuospatial tasks was due to more than just difficulty manipulating a pen or pencil. When correlations between the limb apraxia score and measures of visuospatial performance (ACE-R visuospatial subscore, “written” component) were performed, a strong and highly significant correlation between the overall limb apraxia score and the visuospatial ACE-R subscore (Corr. Co.  = −0.77, P = 0.000) was detected. Even when the “written” components were excluded, a strong and highly significant correlation was detected between the overall limb apraxia score and the “visual” component of the ACE-R visuospatial subtask (Corr. Co.  = −0.64, P = 0.006) was detected. Both correlations survived Bonferroni threshold correction. Separately, there were no significant correlations between either the overall limb apraxia score, or the visuospatial subscore of the ACE-R, and age at symptom onset or symptom duration. In addition, a correlation between the overall limb apraxia and the memory subscore of the ACE-R was detected (Corr. Co.  = −0.619, P = 0.008), and survived Bonferroni correction. No other correlations between the overall limb apraxia score and ACE-R subscores (e.g. language) survived the Bonferroni correction.

### Cortical function

**Figure 2:**
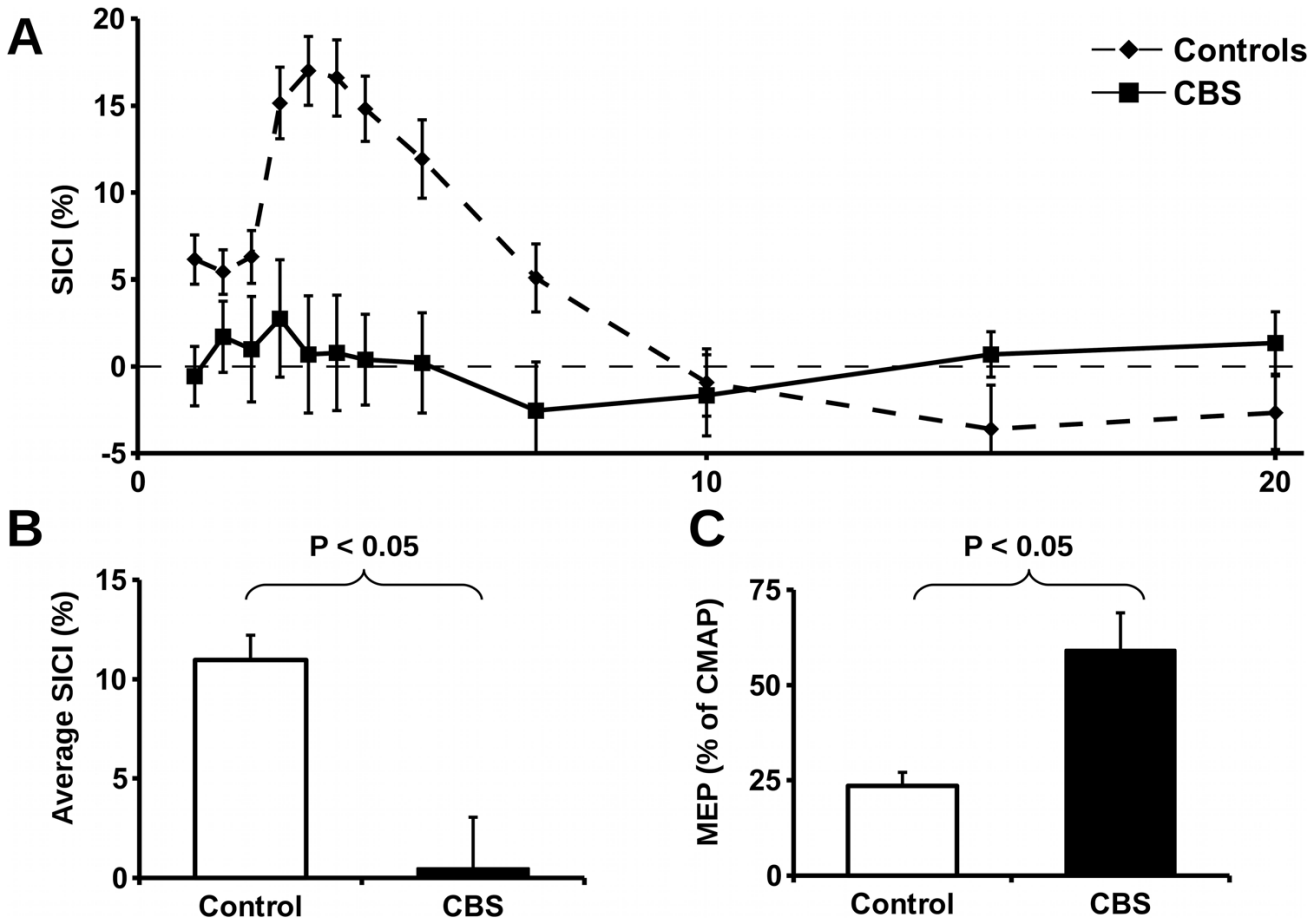
Cortical hyper-excitability in corticobasal syndrome. Patients with CBS had evidence of cortical hyper-excitability, with reduced SICI (**A**), significantly (P<0.05) reduced average SICI (**B**), and significantly (P<0.05) increased motor evoked potential – expressed as a ratio of compound motor action potential amplitude (**C**). Abbreviations: CBS  =  corticobasal syndrome, SICI  =  short-interval intracortical inhibition, MEP  =  motor evoked potential, CMAP  =  compound motor action potential.

Cortical function as assessed by transcranial magnetic stimulation was markedly abnormal in CBS patients with two main patterns of dysfunction. Firstly, 4 (23.5%) had a relatively inexcitable motor cortex (Table 3), which meant that neurophysiological parameters (i.e. RMT, SICI, etc.) could not be measured in these patients. In contrast, only one control subject had a relatively inexcitable motor cortex. Meanwhile, prominent cortical abnormalities were detected among the remaining 76.5% of patients who had an excitable motor cortex. Specifically, 5 (38.5%) CBS patients with an excitable cortex had a reduced RMT (<50%), compared to 1 (5.9%) control (P<0.05). In addition, peak (CBS 0.8+/−12.0, controls 16.6+/−8.7; P<0.001) and average SICI (CBS 0.5+/−9.2, controls 11.0+/−4.9; P<0.001) were significantly reduced in the CBS group compared to controls (Figure 2A and B). The maximum motor evoked potential amplitude (CBS 4.2+/−2.0, controls 1.8+/−1.2; P<0.001) and the motor evoked potential amplitude when expressed as a percentage of compound motor action potential amplitude (CBS 59.1+/−32.6%, controls 23.5+/−14.3; P<0.001) were both increased in CBS compared to controls (Figure 2C). Importantly, there were no significant differences between RMT, peak SICI, or average SICI when the hand used for transcranial magnetic stimulation studies was the most apraxic or the least apraxic hand.

**Table 3:** Cortical excitability in CBS patients.

	CBS	Control	P value
RMT (mean, %)	54.9+/−16.8	60.4+/−8.4	NS
- In-excitable	4 (23.5%)	1 (5.9%)	
- <50%	5 (29.4%)	1 (5.9%)	<0.05*
- >50%	8 (47.1%)	15 (88.2%)	
MEP amplitude (mV)	4.2+/−2.0	1.8+/−1.2	<0.001
MEP amplitude (%)	59.1+/−32.6	23.5+/−14.3	<0.001
Average SICI (%)	0.5+/−9.2	11.0+/−4.9	<0.001
Peak SICI (%)	0.8+/−12.0	16.6+/−8.7	<0.001
CMCT (ms)	7.1+/−0.7	5.8+/−1.8	<0.05
Maximum CSP (ms)	198.6+/−48.0	213.0+/−26.5	NS

Some CBS patients had a relatively inexcitable motor cortex and transcranial magnetic stimulation measures could not be determined. The remaining CBS patients had evidence of cortical excitability, characterized by reduced peak and average SICI, and increased motor evoked potential amplitude expressed as a percentage of compound motor action potential amplitude. CBS  =  corticobasal syndrome, RMT  =  resting motor threshold, SICI  =  short-interval intra-cortical inhibition, MEP  =  motor evoked potential, CSP  =  cortical silent period. *P-value calculated using the Chi-Square test for 2×3 table (i.e. CBD/Control v Inexcitable/<50%/>50%)

### The relationship of cortical dysfunction to clinical features

The CBS cohort was grouped according to the RMT (RMT <50%, RMT >50%, and relatively inexcitable, see *Methods*) and inter-group comparisons were performed (Table 4 and Table S4), albeit with small numbers of patients in each group (a limitation of the present study). Initially, there appeared to be no differences between groups in age, symptom duration, motor weakness or functional impairment (as assessed by the ALSFRS-R). Correlations between the overall limb apraxia score and neurophysiological markers of motor system dysfunction (RMT, Average SICI, motor evoked potential amplitude expressed as a percentage of compound motor action potential amplitude) were attempted with relatively inexcitable patients excluded. A trend was detected for a correlation between reduced RMT and the overall apraxia score (Corr. Co.  = −0.64, P = 0.018), but this did not reach significance after Bonferroni correction for multiple comparisons. This trend (P = 0.07) remained even when partial correlation was used to control for age and symptom duration.

**Table 4:** Clinical features of CBS patients when grouped according to resting motor threshold.

	RMT <50%	RMT >50%	Inexcitable	P -Value
Number of patients	5	8	4	
Symptom Duration (months +/− SD)	54.8+/−13.1	59.3+/−22.6	45.0+/−11.5	NS
Age (years +/−SD)	62.4+/−6.3	64.3+/−5.9	67.3+/−8.7	NS
MRCSS Total	60.0+/−0.0	59.5+/−1.4	60.0+/−0.0	NS
Hyperreflexia (% patients)	4 (80%)	6 (75%)	2 (50%)	NS
**Apraxia**				
- Orobuccal Apraxia (0–3)	0.7+/−0.8	0.2+/−0.4	1.5+/−1.3	NS
- Limb-Meaningful (0–6)	3.9+/−2.2	2.8+/−1.5	4.4+/−1.6	NS
- Limb-Meaningless (0–6)	4.5+/−1.9	3.3+/−1.8	3.9+/−2.3	NS
- Overall Apraxia Score (0–15)	9.1+/−4.2	5.9+/−2.6	9.4+/−4.6	NS
**ALSFRS-R**				
- Bulbar	11.4+/−0.9	10.4+/−1.6	10.3+/−1.3	NS
- Fine Motor	5.2+/−3.3	6.6+/−3.0	4.5+/−4.7	NS
- Gross Motor	8.4+/−2.9	8.9+/−2.6	7.8+/−4.2	NS
- Respiratory	12.0+/−0.0	11.6+/−0.7	12.0+/−0.0	NS
- Total	37.0+/−5.7	37.5+/−5.7	34.5+/−9.0	NS
**ACE-R**				
- Attention	8.8+/−6.4	15.0+/−4.7	14.5+/−5.1	0.09^a^
- Memory	7.8+/−7.9	18.9+/−7.6	18.3+/−6.8	0.06^a,b^
- Fluency	5.8+/−5.2	6.4+/−3.5	3.3+/−5.3	NS
- Language	13.0+/−9.4	19.3+/−7.9	18.3+/−3.0	NS
- Visuospatial	4.2+/−3.8	12.0+/−3.9	10.0+/−4.4	<0.05^a,b^
- Total	38.4+/−31.7	71.5+/−25.6	64.3+/−19.2	NS

Although there were no differences in patient age, symptom duration, limb weakness, or limb functional capacity, patients with a an RMT <50% were significantly more impaired on the visuospatial subtask of the ACE-R, with a trend for impaired performance on the attention and memory ACE-R sub-tasks. CBS  =  corticobasal syndrome, RMT  =  resting motor threshold, MRCSS  =  medical research council sum score, ALSFRS-R  =  amyotrophic lateral sclerosis functional rating score – revised, ACE-R =  Addenbrooke's cognitive examination – Revised, MMSE  =  mini-mental status examination. Note: P-Values quoted in the right hand column refer to inter-group comparisons. Post-hoc pairwise comparisons are indicated by: ^a^RMT <50% versus RMT <50%, P<0.05; ^b^RMT <50% versus Inexcitable, P = 0.063.

Although there was no significant inter-group difference, the CBS sub-group with reduced RMT had a markedly reduced ACE-R total, suggesting severe cognitive impairment. Intergroup comparisons revealed a significant (P<0.05) difference in the visuospatial sub-score, with post-hoc pairwise comparisons confirming a significant reduction in the RMT <50% group compared to the RMT >50% group (P<0.05), and a trend for reduced visuospatial sub-score compared to the relatively inexcitable group (P = 0.06). There were also trends for an intergroup difference in performance on the memory and attention ACE-R subscores, and post-hoc analyses revealed that the RMT <50% group had significantly lower memory and attention sub-scores (P<0.05) compared to the RMT >50% group.

### Neuroanatomical correlates of functional and clinical characteristics

**Figure 3:**
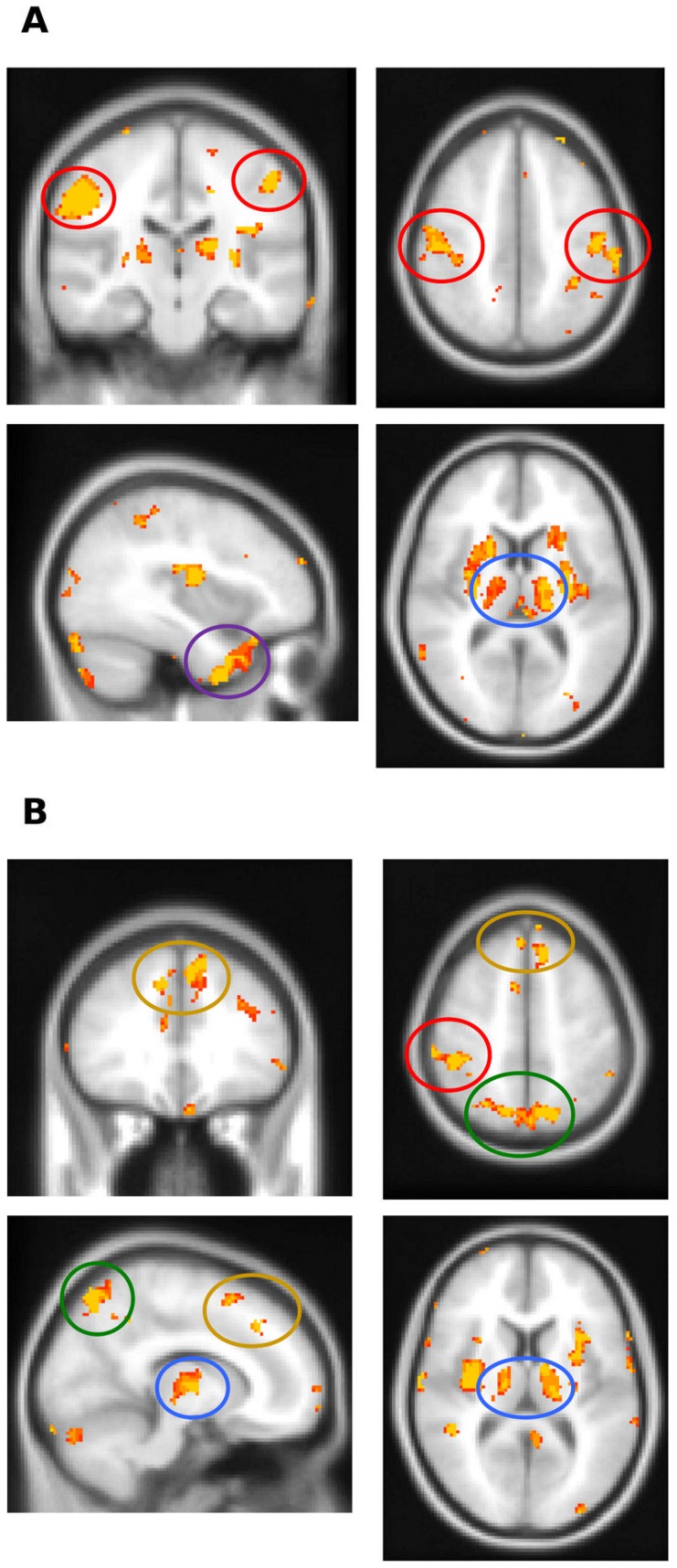
Voxel-based morphometry analysis demonstrating brain regions that positively correlate with neurophysiological parameters in CBS patients. (A) Reduced RMT correlated with atrophy of the primary motor cortex (red circles), thalamus (blue circle) and the anterior temporal lobe (magenta circle). (B) Reduced SICI correlated with atrophy of the primary motor cortex (red circle), thalamus (blue circles), medial frontal cortex (yellow circles) and precuneus (green circles). Clusters are overlaid on the Montreal Neurological Institute standard brain (t>2.41). Colored voxels show regions that were significant in the analyses for P<0.001 uncorrected and a cluster threshold of 20 contiguous voxels. Circled areas indicate: red  =  primary motor cortex; blue  =  thalamus; magenta  =  anterior temporal lobe; yellow  =  medial frontal cortex; green  =  precuneus.

**Figure 4:**
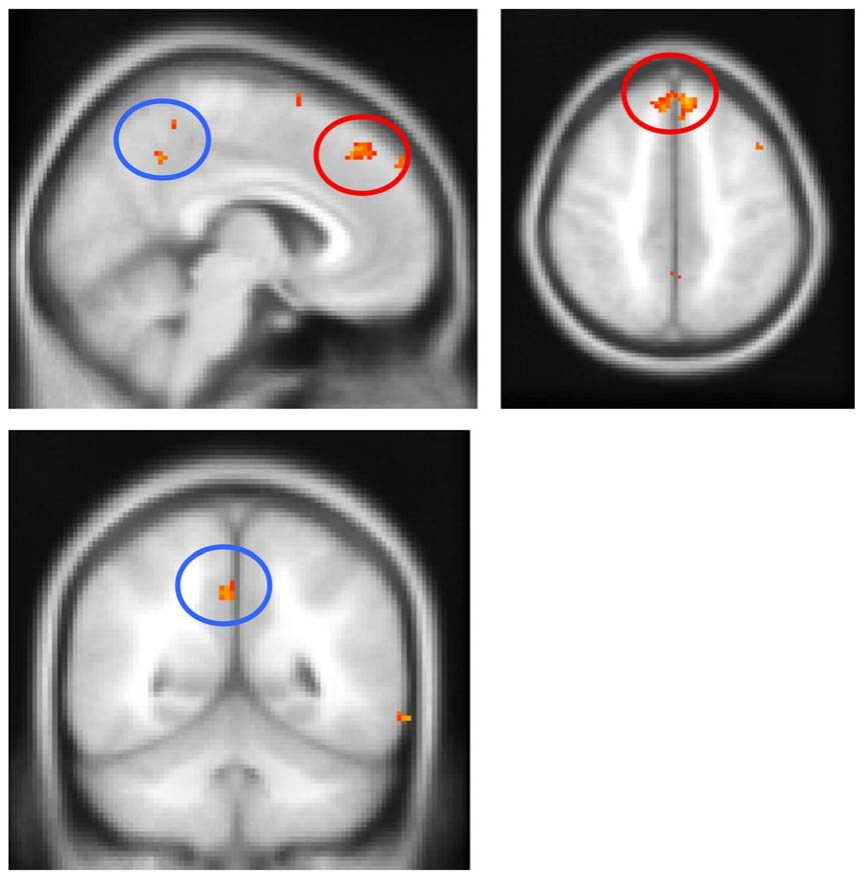
Voxel-based morphometry analysis with the apraxia score as a covariate in CBS patients. The degree of apraxia (as reflected in an increased apraxia score) correlated with atrophy of the medial frontal cortex (red circles) and the precuneus/posterior cingulate (blue circles). Clusters are overlaid on the Montreal Neurological Institute standard brain (t>2.41). Colored voxels show regions that were significant in the analyses for P<0.001 uncorrected and a cluster threshold of 20 contiguous voxels. Circled areas indicate: red  =  medial frontal cortex; blue  =  precuneus/posterior cingulate.

To further clarify the basis of cortical dysfunction, VBM analyses were performed using transcranial magnetic stimulation excitability measures (RMT, SICI) and the overall apraxia score. Since RMT and SICI could not be measured in patients with a relatively inexcitable motor cortex, these cases were excluded from VBM analyses using SICI and RMT as covariates. Importantly, visual inspection of MRI scans did not reveal differences in the degree or pattern of atrophy in cortical and subcortical motor structures between patients with a relatively inexcitable motor cortex, and those in whom SICI and RMT could be measured. All CBS patients were included in the overall apraxia score VBM analysis. As demonstrated in Figure 3, reduced RMT and SICI correlated with atrophy of the primary motor cortex, as well as the basal ganglia and thalamus bilaterally. In addition, reduced RMT correlated with atrophy of the pre-central gyrus, insula and left anterior temporal lobe. Similarly, reduced SICI correlated with insula, left medial frontal cortex and bilateral precuneus atrophy. Finally, the degree of apraxia, indicated by an increased overall apraxia score, correlated with atrophy of the medial frontal cortex bilaterally, as well as the precuneus and posterior cingulate brain regions (Figure 4).

## Discussion

The present study has demonstrated significant cortical dysfunction in a cohort of CBS patients. VBM established a correlation between measures of cortical dysfunction and atrophy of the primary motor cortex, as well as subcortical motor structures. A relationship between the distinctive features of CBS, namely limb apraxia and visuospatial dysfunction, was demonstrated and limb apraxia was linked to motor system dysfunction, as measured by transcranial magnetic stimulation. Altogether, the findings from the present study reinforce the role of frontal lobe (i.e. primary motor cortex) dysfunction in the development of the characteristic features of CBS.

Cortical dysfunction was indicated in the present series by a high proportion of CBS patients exhibiting an RMT <50%, reduced SICI, and an increased motor evoked potential amplitude. Although reduced SICI has been suggested in CBS previously [12]–[15], [57], reported changes in RMT have been inconsistent. Some studies have demonstrated increased RMT,[12], [13], [57], [58] while others reported no change.[15] A possible explanation for these discordant findings is that previous studies compared mean RMT values, unlike the present approach which grouped patients according to their RMT; one benefit of this approach is that the distribution of RMT levels across the entire CBS cohort can be taken into account. One limitation of the present study was that side-to-side comparisons of cortical function were not performed. As such, the relationship of any asymmetry in responses to clinical features or underlying pathology is currently unknown.

The mechanisms underlying cortical dysfunction in CBS remain to be fully elucidated. RMT reflects the excitability of cortical motor neurons,[59], [60] but SICI reflects the function of inhibitory intra-cortical circuits acting via GABAa receptors.[36], [60], [61] Degeneration of inhibitory cortical inter-neurons may therefore account for the transcranial magnetic stimulation results, particularly since reduced RMT and SICI correlated with atrophy of the primary motor cortex. In addition, disinhibition of the motor cortex by dysfunctional basal ganglia and thalamus may have contributed.[12]

Why some patients with CBS have a relatively inexcitable motor cortex while others have evidence of reduced RMT and SICI is not clear, but may relate to different underlying pathologies. Given that there were no differences between patients when grouped by RMT in symptom duration, the degree or pattern of cortical atrophy, motor weakness or functional impairment, advanced disease stage alone is unlikely to explain the finding of a relatively inexcitable motor cortex in CBS. Moreover, the relatively inexcitable group was not the most cognitively impaired. In contrast, the RMT <50% group demonstrated the greatest cognitive impairment, with poorer performance on visuospatial, attention, memory, and overall ACE-R scores. Poor performance on the attention and memory ACE-R subtasks in CBS has been linked to underlying Alzheimer's disease [4] and visuospatial dysfunction may be even more severely affected in CBS cases due to Alzheimer's disease than in other subtypes.[23], [54] Finally, RMT has been demonstrated to be significantly reduced in Alzheimer's disease.[62] As such, findings from the present series may suggest that transcranial magnetic stimulation measures of cortical dysfunction in CBS are related to underlying pathology. However, the number of patients with an inexcitable cortex or reduced RMT in the present study is relatively small, therefore larger studies comparing cortical function across pathologically defined CBS sub-groups will be required to confirm this possibility.

Apraxia assessment tools, for example the De Renzi ideomotor apraxia test,[63] have been designed and published previously, although no single instrument has been widely adopted. Many of the previously reported assessment tools have attempted to document the various sub-types of apraxia and may be lengthy and time-consuming as a result. This may render them impractical for routine clinical use.[64] Given that the focus of the present study was to examine the relationships between limb apraxia and motor system dysfunction, rather than on the sub-types of apraxia per se, a simple but robust grading system based on imitation of meaningless and meaningful hand gestures was used, rather than using a more detailed instrument; this could be viewed as a limitation of the present study. Nonetheless, our grading of apraxia severity was simple and easy to apply, and related closely to the level of functional impairment as assessed by the ALSFRS-R. As such, our assessment was brief enough to use in the clinic, but still appeared to yield useful, clinically relevant, information.

Several sub-types of apraxia have been described in CBS, including oro-buccal, limb-kinetic, ideomotor (i.e. temporal or spatial errors in goal-directed movements),[65] ideational (i.e. impaired sequencing of tasks) [5] and conceptual (failure to use tools correctly).[17], [29], [30] More significant impairment of transitive gestures, rather than intransitive gestures, has also been reported in previous studies.[17], [30] The clinical and diagnostic validity of such complex sub-classifications has not been established in the context of CBS. Another challenge is to differentiate any functional impairment due to dystonia and rigidity, from that due to apraxia. As such, apparent asymmetry in apraxia scores in the present study may have reflected superimposed dystonia or rigidity rather than just limb apraxia.

In the present study the degree of apraxia, reflecting impaired imitation, was strongly correlated with performance on visuospatial tasks, regardless of whether the task required manipulation of a pencil or simply the interpretation of visual information. Similarly, a correlation between impaired imitation of hand movements and visuospatial dysfunction has been suggested in Parkinson's disease.[66] These results need to be interpreted with caution; it is entirely possible that deficits in visual processing are responsible for impaired imitation, rather than apraxia per se. On the other hand, the correlation between limb apraxia and visuospatial impairment does not confirm causality between the two deficits. Although the precise relationship between limb apraxia and visuospatial dysfunction remains to be elucidated, the results from the present study suggest that limb apraxia could be partly due to impaired visuospatial processing or visuomotor transcoding.[67] Whether this relationship extends to aspects of apraxia not tested through imitation remains unknown. Separately, difficulties in imitation may need to be considered when designing and administering physical therapies in patients with CBS.

The effect of asymmetric pathological involvement on apraxia and visuospatial dysfunction in CBS is also unknown. In the context of stroke, limb apraxia is much more likely after a left-sided than a right-sided stroke.[68] In the present study, 7 patients had symmetrical atrophy, whereas 10 had asymmetric atrophy (left > right in all but one patient). No significant differences in the visuospatial ACE-R or the apraxia subscore were detected between the two groups. Unlike stroke patients, the locus of pathology in CBS is virtually always bilateral, even when atrophy is asymmetric. Nonetheless, in the presence of severe cortical atrophy, both the right and left hemispheres may be affected, making the distinction between left and right parietal contributions to symptomatology very difficult to establish.

A clear consensus on the neuroanatomical basis of apraxia in CBS has not yet emerged, perhaps reflective of the complex neural networks involved in gesture production and tool usage. Through lesional studies, several cerebral structures have been implicated in the genesis of apraxia, including the left parietal lobe,[17], [18], [69], [70] frontoparietal circuits,[21] and the premotor cortex.[71]–[73] Apraxia has also been described in the context of basal ganglia pathology, particularly if surrounding white matter tracts are involved.[74] In CBS, pathological studies have attributed apraxia to involvement of the parietal, premotor, and primary motor cortices.[23], [71], [75]–[77] Imaging studies in CBS have correlated apraxia with inferior parietal,[21], [22] left supplementary motor area, premotor cortex, and caudate nucleus atrophy or dysfunctional connectivity between these regions.[21], [29] The parietal lobe is important for integration of visual and sensory information,[78] and involvement of this key integrative region in CBS is well recognized, particularly in CBS associated with underlying Alzheimer's disease.[23], [24] Our analyses suggest a strong relationship between apraxia and atrophy of two regions; the precuneus/posterior cingulate within the posteromedial parietal lobe, and the premotor cortex. Interestingly, functional MRI studies also support a key role for these two regions.[79] The precuneus is known to be affected early in the course of Alzheimer's disease,[80] suggesting that the group of CBS patients with underlying Alzheimer's pathology may be driving the association. Future studies should use amyloid imaging techniques (such Pittsburgh compound type B positron emission tomography) to explore this relationship. Furthermore, the precuneus/posterior cingulate should be examined more closely in CBS patients with underlying tau pathology.

In summary, cortical dysfunction in CBS is associated with pathological involvement of the primary motor cortex and the basal ganglia. Motor dysfunction, as assessed by transcranial magnetic stimulation techniques, was associated with the degree of apraxia. In addition, limb apraxia was correlated with atrophy of the precuneus and the pre-motor cortex. Comparisons of cortical excitability across different pathologies in CBS are required to determine whether transcranial magnetic stimulation may be useful in predicting pathology in life. Nonetheless, by combining functional neurophysiological and neuropsychological methods, as well as VBM, the present study provides further insight into the pathogenesis of core clinical features of CBS.

## Supporting Information

Table S1 **Inter-rater reliability for measures of apraxia.** Mean apraxia scores did not significantly differ when examiner 1 was compared to examiner 2.
(DOC)

Table S2 **Motor features of corticobasal syndrome patients.** Motor dysfunction in CBS patients was characterised by rigidity and bradykinesia, while other features such as alien limb phenomenon, myoclonus, and dystonia were less common.
(DOCX)

Table S3 **Individual performance of CBS patients on the ACE-R.** Performance in the impaired range (Mioshi et al., 2006) is indicated by highlighted cells according to age defined cut-offs (yellow  = 50–59 years, purple  = 60–69 years, orange  = 70–75 years). Note the ACE-R could not be performed on one patient.
(DOCX)

Table S4 **Motor features of corticobasal syndrome patients classified by resting motor threshold.** Motor dysfunction in CBS patients was characterised by rigidity and bradykinesia, while other features such as alien limb phenomenon, myoclonus, and dystonia were less common.
(DOCX)
