# Supplementary material for: Apraxia and Motor Dysfunction in Corticobasal Syndrome
Source: PLoS One. 2014 Mar 24;9(3):e92944. doi: 10.1371/journal.pone.0092944 (PMC3963965; doi:10.1371/journal.pone.0092944)
Supplement: Table S1 — Inter-rater reliability for measures of apraxia. Mean apraxia scores did not significantly differ when examiner 1 was compared to examiner 2. (DOC) [file pone.0092944.s001.doc]

**Table S1**: ***Inter-rater reliability for measures of apraxia.*** Mean apraxia scores did not significantly differ when examiner 1 was compared to examiner 2.

| **APRAXIA SCORE** |  | **P Value** |
| --- | --- | --- |
| **Meaningful Gestures** (mean +/- SD) |  |  |
| Examiner 1 | 3.5 +/- 1.7 | NS |
| Examiner 2 | 3.8 +/- 1.9 |  |
| **Meaningless Gestures** (mean +/- SD) |  |  |
| Examiner 1 | 4.1 +/- 1.8 | NS |
| Examiner 2 | 3.6 +/- 2.1 |  |
| **Overall Apraxia Score** (mean +/- SD) |  |  |
| Examiner 1 | 8.1 +/- 3.7 | NS |
| Examiner 2 | 7.7 +/- 4.3 |  |
