# Supplementary material for: Apraxia and Motor Dysfunction in Corticobasal Syndrome
Source: PLoS One. 2014 Mar 24;9(3):e92944. doi: 10.1371/journal.pone.0092944 (PMC3963965; doi:10.1371/journal.pone.0092944)
Supplement: Table S2 — Motor features of corticobasal syndrome patients. Motor dysfunction in CBS patients was characterised by rigidity and bradykinesia, while other features such as alien limb phenomenon, myoclonus, and dystonia were less common. (DOCX) [file pone.0092944.s002.docx]

**Table S2: *Motor features of corticobasal syndrome patients***. Motor dysfunction in CBS patients was characterised by rigidity and bradykinesia, while other features such as alien limb phenomenon, myoclonus, and dystonia were less common.

| Number of patients | 17 |
| --- | --- |
| Myoclonus (%) | 3 (17.6%) |
| Alien Limb Phenomenon (%) | 3 (17.6%) |
| Postural Instability (%) | 9 (52.9%) |
| Gait Disturbance (%) |  |
| Dystonia (%) | 5 (29.4%) |
| **Tremor** |  |
| Right (%) | 2 (11.8%) |
| Left (%) | 1 (5.9%) |
| **Rigidity** |  |
| Right (%) | 14 (82.4%) |
| Left (%) | 11 (64.7%) |
| **Bradykinesia** |  |
| Right (%) | 15 (88.2%) |
| Left (%) | 13 (76.5%) |
