# Supplementary material for: Apraxia and Motor Dysfunction in Corticobasal Syndrome
Source: PLoS One. 2014 Mar 24;9(3):e92944. doi: 10.1371/journal.pone.0092944 (PMC3963965; doi:10.1371/journal.pone.0092944)
Supplement: Table S3 — Individual performance of CBS patients on the ACE-R. Performance in the impaired range (Mioshi et al., 2006) is indicated by highlighted cells according to age defined cut-offs (yellow = 50–59 years, purple = 60–69 years, orange = 70–75 years). Note the ACE-R could not be performed on one patient. (DOCX) [file pone.0092944.s003.docx]

**Table S3: *Individual performance of CBS patients on the ACE-R***.

Performance in the impaired range (Mioshi et al., 2006) is indicated by highlighted cells according to age defined cut-offs (yellow = 50-59 years, purple = 60-69 years, orange = 70-75 years). Note the ACE-R could not be performed on one patient.

|  | Attention/  Orientation | Memory | Fluency | Language | Visuospatial | Total |
| --- | --- | --- | --- | --- | --- | --- |
| 56 | 5 | 3 | 2 | 13 | 2 | 25 |
| 57 | 13 | 11 | 8 | 16 | 5 | 53 |
| 57 | 14 | 20 | 5 | 24 | 14 | 77 |
| 58 | 18 | 25 | 11 | 17 | 8 | 79 |
| 59 | 18 | 20 | 12 | 24 | 12 | 86 |
| 59 | 18 | 24 | 6 | 25 | 13 | 86 |
| 61 | DK | DK | DK | DK | DK | DK |
| 62 | 18 | 25 | 4 | 23 | 14 | 84 |
| 65 | 10 | 8 | 5 | 9 | 6 | 38 |
| 66 | 17 | 14 | 0 | 19 | 12 | 62 |
| 66 | 7 | 11 | 0 | 15 | 5 | 38 |
| 68 | 18 | 23 | 9 | 25 | 16 | 91 |
| 68 | 16 | 20 | 12 | 26 | 10 | 84 |
| 70 | 10 | 5 | 1 | 10 | 4 | 30 |
| 72 | 18 | 25 | 9 | 19 | 15 | 86 |
| 72 | 6 | 6 | 1 | 5 | 6 | 24 |
| 79 | 16 | 23 | 2 | 22 | 15 | 78 |
|  |  |  |  |  |  |  |
| % of patients in impaired range | 50% | 44% | 69% | 63% | 81% | 75% |
